# ANANASTRA: annotation and enrichment analysis of allele-specific transcription factor binding at SNPs

**DOI:** 10.1093/nar/gkac262

**Published:** 2022-04-21

**Authors:** Alexandr Boytsov, Sergey Abramov, Ariuna Z Aiusheeva, Alexandra M Kasianova, Eugene Baulin, Ivan A Kuznetsov, Yurii S Aulchenko, Semyon Kolmykov, Ivan Yevshin, Fedor Kolpakov, Ilya E Vorontsov, Vsevolod J Makeev, Ivan V Kulakovskiy

**Affiliations:** Vavilov Institute of General Genetics, Russian Academy of Sciences, Moscow, 119991, Russia; Moscow Institute of Physics and Technology, Dolgoprudny, 141701, Russia; Laboratory of Regulatory Genomics, Institute of Fundamental Medicine and Biology, Kazan Federal University, Kazan, 420008, Russia; Vavilov Institute of General Genetics, Russian Academy of Sciences, Moscow, 119991, Russia; Moscow Institute of Physics and Technology, Dolgoprudny, 141701, Russia; Laboratory of Regulatory Genomics, Institute of Fundamental Medicine and Biology, Kazan Federal University, Kazan, 420008, Russia; Institute of Protein Research, Russian Academy of Sciences, Pushchino, 142290, Russia; Institute of Protein Research, Russian Academy of Sciences, Pushchino, 142290, Russia; Southern Federal University, Rostov-on-Don, 344006, Russia; Moscow Institute of Physics and Technology, Dolgoprudny, 141701, Russia; Institute of Mathematical Problems of Biology RAS - the Branch of Keldysh Institute of Applied Mathematics of Russian Academy of Sciences, Pushchino, 142290, Russia; Skolkovo Institute of Science and Technology, Moscow, 121205, Russia; Institute of Cytology and Genetics SB RAS, Novosibirsk, 630090, Russia; PolyKnomics BV, ’s-Hertogenbosch, 5237 PA, Netherlands; Sirius University of Science and Technology, Sochi, 354340, Russia; Biosoft.Ru LLC, Novosibirsk, 630090, Russia; Sirius University of Science and Technology, Sochi, 354340, Russia; Biosoft.Ru LLC, Novosibirsk, 630090, Russia; Sirius University of Science and Technology, Sochi, 354340, Russia; Federal Research Center for Information and Computational Technologies, Novosibirsk, 630090, Russia; Vavilov Institute of General Genetics, Russian Academy of Sciences, Moscow, 119991, Russia; Institute of Protein Research, Russian Academy of Sciences, Pushchino, 142290, Russia; Vavilov Institute of General Genetics, Russian Academy of Sciences, Moscow, 119991, Russia; Moscow Institute of Physics and Technology, Dolgoprudny, 141701, Russia; Laboratory of Regulatory Genomics, Institute of Fundamental Medicine and Biology, Kazan Federal University, Kazan, 420008, Russia; Engelhardt Institute of Molecular Biology, Russian Academy of Sciences, Moscow, 119991, Russia; Vavilov Institute of General Genetics, Russian Academy of Sciences, Moscow, 119991, Russia; Laboratory of Regulatory Genomics, Institute of Fundamental Medicine and Biology, Kazan Federal University, Kazan, 420008, Russia; Institute of Protein Research, Russian Academy of Sciences, Pushchino, 142290, Russia

## Abstract

We present ANANASTRA, https://ananastra.autosome.org, a web server for the identification and annotation of regulatory single-nucleotide polymorphisms (SNPs) with allele-specific binding events. ANANASTRA accepts a list of dbSNP IDs or a VCF file and reports allele-specific binding (ASB) sites of particular transcription factors or in specific cell types, highlighting those with ASBs significantly enriched at SNPs in the query list. ANANASTRA is built on top of a systematic analysis of allelic imbalance in ChIP-Seq experiments and performs the ASB enrichment test against background sets of SNPs found in the same source experiments as ASB sites but not displaying significant allelic imbalance. We illustrate ANANASTRA usage with selected case studies and expect that ANANASTRA will help to conduct the follow-up of GWAS in terms of establishing functional hypotheses and designing experimental verification.

## Graphical Abstract

**Graphical Abstract:**
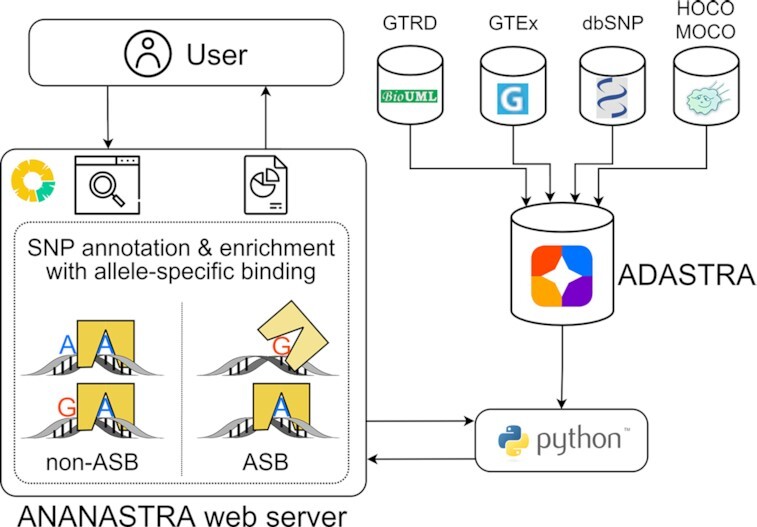
ANANASTRA (https://ananastra.autosome.org) is a web server for annotation and enrichment analysis of allele-specific transcription factor binding at regulatory single-nucleotide polymorphisms (SNPs) in a submitted list of dbSNP IDs or a VCF file.

## INTRODUCTION

In silico functional annotation of single-nucleotide polymorphisms (SNPs) detected in genome-wide association studies (GWAS) is an essential step that facilitates a transition from statistical association between a genome variant and a trait to understanding the biological mechanism of genome-conditioned trait formation (1). Most SNPs found in GWAS are located outside of protein-coding segments and are believed to exhibit their effects through gene regulation, particularly, at the level of transcription, by altering binding affinity of transcription factors.

A multitude of software and resources (2,3), including web servers, are available for computational annotation of a single SNP. A common approach to identify SNPs affecting transcription factor binding is assessment in silico, e.g. by predicting DNA sites binding transcription factors (TF) using classic position weight matrices (4–6), or with the help of traditional machine learning (7) and artificial neural networks (8). Yet, it is desirable to have information on differential TF binding relying on experimental data rather than computational predictions. Such data can be obtained with massive parallel reporter assays (9–11) or in vitro methods such as recently presented SNP-SELEX (12).

For transcription binding variation in vivo, heterozygous sites of homologous chromosomes provide a valuable data source. These sites are often captured in large-scale ChIP-Seq experiments and provide rich, although mostly unstructured data (13–16) on allele-specific binding (ASB), where TF exhibits different binding affinity depending on a particular allele. The ASB sites can be recovered from the wealth of ChIP-Seq data and consequently used for functional annotation of regulatory SNPs of interest. SNPs exhibiting different transcription factor binding affinity to alternative alleles can serve as promising candidates for regulatory SNPs of a functional consequence involved in phenotype formation (17,18). The allele-specific binding data from ChIP-Seq has a special advantage over computational predictions or in vitro assays, as it ensures the transcription factor was not only expressed in the cell type of interest but also showed differential binding to alternative alleles. Yet, up until now, there have been no systematic resources allowing a user to utilize such data conveniently.

We have recently presented ADASTRA, the database of Allelic Dosage-corrected Allele-Specific human TRAnscription factor binding sites (19). ADASTRA provides detailed information on ASB events at particular SNPs. However, in many cases it is more desirable to annotate a large set of variants at once, and, simultaneously, perform a statistical test checking for possible enrichment of allele-specific binding events for particular TFs or cell types.

Here, we present a new web server, ANANASTRA, named for **an**notation and enrichment **an**alysis of **a**llele-**s**pecific **tra**nscription factor binding at SNPs. Atop ‘one-click’ annotation of multiple user-submitted SNPs, ANANASTRA performs general enrichment analysis for ASBs, also checking particular TFs and cell types.

## MATERIALS AND METHODS

### Overview of the workflow

**Figure 1.**
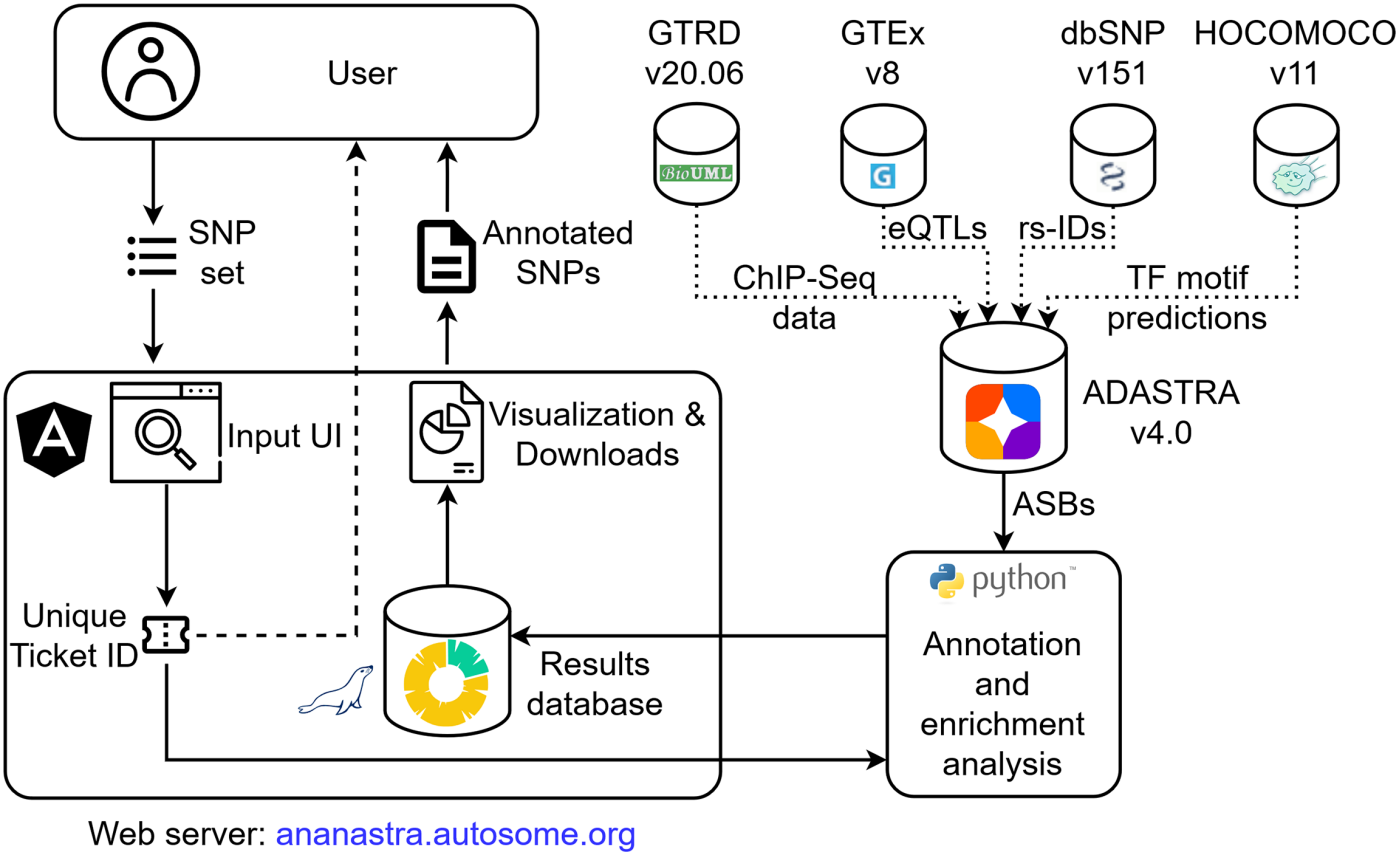
Schematic representation of the ANANASTRA workflow. ANANASTRA accepts a list of dbSNP IDs, generates a unique job Ticket ID, and returns it to the user. The Ticket ID provides the access to the annotation of the SNPs with allele-specific binding events and the enrichment analysis results. The underlying data is taken from ADASTRA which internally utilizes ChIP-Seq data from GTRD and matches allele-specific events against GTEx eQTLs and HOCOMOCO sequence motifs.

The general workflow of the analysis is presented in Figure 1. A user submits a list of SNPs of interest either in plain text (one dbSNP (20) rsID per line) or as a standard VCF file (based on hg38 genome assembly (21)). In both cases, the contents can be copy-pasted into the form or uploaded (gzipped VCF is supported). In a single run, ANANASTRA performs two types of analysis: (i) annotates the submitted list of SNPs with allele-specific binding and (ii) checks if the ASBs are enriched in general or considering particular TFs or cell types as compared to the predefined background. To playtest the service, a user may use built-in example data (see case studies below). The user set options are (a) FDR threshold for considering the sites as ASBs (defaults to 5%) and (b) background, which can be either ‘local’ (default, background SNPs are drawn from merged 1Mb-windows centered on the user-submitted SNPs), based on linkage disequilibrium islands (LD-islands) estimated for three populations in (22), or whole-genome (generally not recommended as it does not account for non-uniform distribution of ASBs in different genomic regions). Upon form submission, the system puts the job into a queue and generates a unique ‘Ticket ID’ that allows checking the status of the job and accessing the results upon completion of the job.

### Annotation of SNPs

ANANASTRA uses ADASTRA data on ASBs, including both transcription factor (TF)-centric and cell type-centric information. For a given SNP, TF-ASBs reflect statistically significant preferential binding observed in the whole set of ChIP-Seq experiments for particular transcription factors. Cell type-ASBs reflect significant preferential binding of various TFs in the selected cell type. For both TF- and cell type-ASBs, there are data on preferred binding to the reference allele (Ref-ASBs) and alternative allele (Alt-ASBs) based upon hg38 genome assembly. A single SNP can display both Ref- and Alt- ASB e.g. for different TFs or cell types. Both TF- and cell type-ASB annotations are performed by ANANASTRA. FDR-passing entries at SNPs of interest are either displayed separately for each TF (‘Expanded’ view) or grouped by SNP rsID with a single top-significant entry being shown (‘Collapsed’ view). An interactive table and barplot allow sorting and filtering of the resulting lists. Detailed information on individual ChIP-Seq experiments supporting a particular ASB event is available by clicking the corresponding table row. Only FDR-passing entries are included in the online report page. Additionally, significant ASBs are linked with GTEx eQTLs and, for TF-ASBs, checked for concordance with HOCOMOCO motifs. Complete data on the ASB sites (passing user-defined FDR threshold), non-ASB sites (with FDR above 25% where ASBs are unlikely), and sites with undefined ASB status (with FDR in between) are available in ‘Downloads’. The tables of TF-ASBs and cell type-ASBs are available for download in both ‘Expanded’ and ‘Collapsed’ forms. Additionally, in ‘Downloads’ we provide a list of ASB-supported eQTL target genes, which can be used for the downstream analysis, e.g. GO-term enrichment.

### Enrichment analysis

To perform enrichment analysis, ANANASTRA utilizes a one-sided Fisher's exact test. The test compares the numbers of SNPs with significant ASBs and without significant ASBs in the user-submitted SNP list against the SNPs with similar ChIP-Seq coverage located in background regions. Notably, the sites with an undefined ASB status (with the FDR in between 0.25 and the user-defined threshold) are excluded from the test and only candidate ASB sites passing the same read coverage thresholds (see Abramov et al. (19) for details) are considered in the positive and background sets. Enrichment is estimated for SNPs with any ASBs, or specifically TF- or cell type-specific ASBs. A donut chart displaying the distribution of SNPs across ASB annotation categories together with ASB enrichment statistics is provided at the top of the summary tab of the report page. Additionally, in the top section of each report page there are barplots illustrating the results of enrichment analysis along with the underlying table data.

### The underlying data and updates

The ANANASTRA release described in this paper is based on ADASTRA v4.0 (release Zanthar), which utilizes dbSNP 151, GTEx v8, GTRD v20.06 and HOCOMOCO v11. We plan to maintain ANANASTRA and update it along with ADASTRA. Thus, the case studies used as examples in this paper can receive different annotation and/or enrichment estimates in the future. Static report pages for the case studies based on the current ANANASTRA release are persistently available with ticket IDs ‘example1’ and ‘example2’.

### Web server implementation

The web interface is implemented as an Angular application. Once the user input is validated, a new job is put into the scheduler queue. The Python backend module annotates submitted SNPs with information from ADASTRA, HOCOMOCO, and GTEx databases. The results are stored in the internal MySQL database for 72 hours after submission and are accessible via a unique Ticket ID.

SNP sets of up to 10000 entries are accepted for online processing. The same limits apply to an SNP list extracted from the user-uploaded VCF file, the upload file size limit is 100 kilobytes and applies to gzipped files as well. In the case of larger VCF files and SNP sets, we invite the users to contact for the special arrangement to process a larger job.

ANANASTRA uses the HTTPS protocol, includes a help page with a glossary, example data sets (directly at the landing page), and interactive page tours explaining the analysis reports. For convenience, when processing user requests, ANANASTRA randomly assigns unique ‘Ticket IDs’ that allow re-accessing the results while ensuring user data privacy.

For programmatic access to ANANASTRA we provide REST API (https://ananastra.autosome.org/api/v4).

## RESULTS

To illustrate the practical applicability of ANANASTRA, we designed two case studies, which are available as demonstration examples 1 and 2 on the web server landing page.

### Case study 1: Annotating a credible set of SNPs associated with inflammatory bowel disease

**Figure 2.**
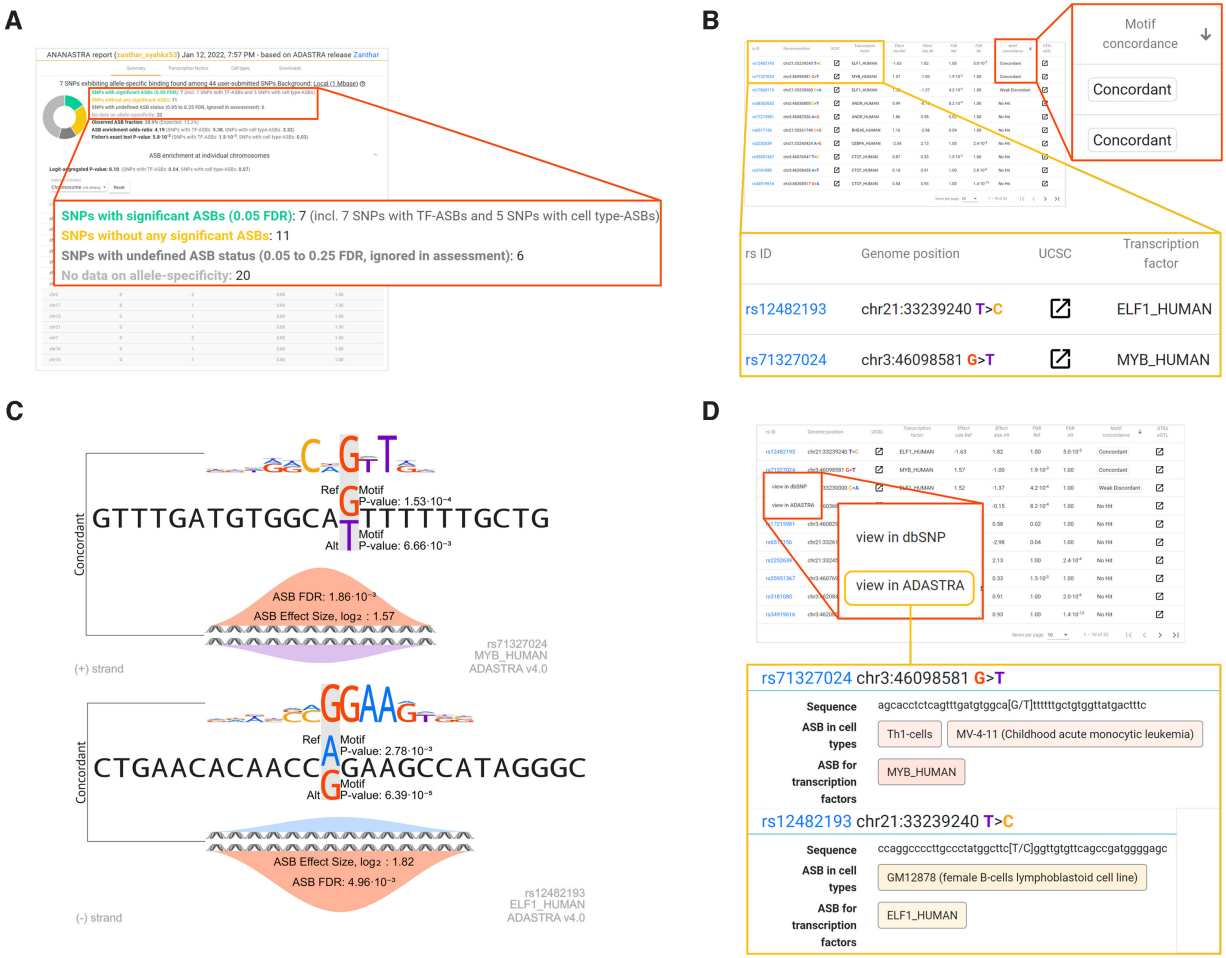
Highlights of the ANANASTRA case studies. (**A**) General enrichment of ASBs in the credible set of SNPs associated with inflammatory bowel disease (Example 1 on the website). (**B**) Motif-concordant ASBs found among the top significant SNPs of COVID19-hg GWAS meta-analyses (Example 2 on the website): rs12482193 (T/C) and rs71327024 (G/T). (**C**) Motif annotation and ASB effect size illustration of rs12482193 for ELF1 and rs71327024 for MYB of COVID19-hg GWAS meta-analyses (Example 2 on the website). Note, that rs12482193 is shown in the reverse complementary orientation (-strand) to match the motif logo visualization. The bells at the bottom subpanels of each illustration correspond to the alleles preferentially bound by the respective transcription factors in vivo. (**D**) Accessing the ASB details for rs12482193 and rs71327024 in ADASTRA.

Regulatory SNPs are deeply involved in hereditary genetics of complex diseases including various autoimmune disorders (23). As a first case study, we took the credible set of SNPs associated with inflammatory bowel disease (24). This case study is accessible as Example 1 on the landing page. Table 1 of the work (24) lists SNPs with posterior probability > 50% of being causal. Of the 44 dbSNP IDs listed in the Table, seven coincide with ASBs (Figure 2A). The absolute number of ASB SNPs is small. Still, the relative enrichment against the local background is over 4-fold (*P* < 0.01), indicating a significant overrepresentation of allele-specific binding events among the SNPs from the credible set. Notably, four of five SNPs with cell type-ASBs and six of seven SNPs with TF-ASBs are significant GTEx eQTLs.

Considering particular SNPs, there is rs61839660 located in the intron region of the *IL2RA* gene. The downregulated *IL2RA* expression is associated with the development of Crohn's disease (25) and rs61839660-T was previously shown to downregulate *IL2RA* expression by reducing affinity for the MEF2 factors (26). It is known that BRD4 can recruit P-TEFb to transcriptionally active promoters, and overexpression of P-TEFb stimulates MEF2-dependent transcription (27). In agreement with this, rs61839660 coincides with the ASB site for BRD4, with the preference for the reference allele (rs61839660-C).

### Case study 2: Annotating top significant SNPs found in COVID19-hg GWAS meta-analyses

Thanks to the efforts of the COVID-19 Host Genetics Initiative, there are data on individual variants affecting the chance of being infected with SARS-CoV-2 and the severity of COVID-19 (28). These data provide another interesting case study for ANANASTRA. Example 2 on the landing page consists of the top 500 significant SNPs found in the meta-analyses of a total of 24274 hospitalized COVID-19 cases and 2061529 population controls from COVID19-hg GWAS meta-analyses round 6 results (https://www.covid19hg.org/results/r6/).

ANANASTRA annotates significant allele-specific binding events at 31 of 500 SNPs, which is 2-fold enrichment over local background (p < 0.01). There are also two SNPs of particular interest, with TF-ASB events that are concordant with motif annotation (Figure 2B), i.e. where ChIP-Seq allelic imbalance is in full agreement with the sequence-level computational TFBS motif predictions. This means that the TF binding occurs directly at a particular SNP, and the allelic substitution is mechanistically causal for changes of TF binding affinity (Figure 2C).

First, for rs71327024, the affected TF is MYB. Switching to the cell type-information, clicking the respective table row in the TF-centric view, or following the link to the detailed SNP-level information in ADASTRA (Figure 2D), one can discover that the allele-specific binding was observed in T Helper 1 (Th1)-cells. Furthermore, according to GTEx, this SNP serves as an eQTL for chemokine receptors CXCR6 and CCR1. CXCR6 is expressed in T lymphocytes and recruits CD8-resident memory T cells in the airways to fight respiratory pathogens (29). In a recent study, decreased CXCR6 expression was shown to correlate with the severity of COVID-19 (30). CCR1 mediates monocyte/macrophage polarization and tissue infiltration (31). CCR1 is overexpressed in monocytes and neutrophils in COVID-19 (32) and serves as a sign of a severe illness (33).

Another motif-concordant ASB is found for ELF1 TF at rs12482193 (T > C), which is located in the intron region of *IFNAR2* and serves as an eQTL for *IFNAR2* and *IL10RB*. *IFNAR2* encodes one of the two chains of the IFNα/β receptor. Reduced expression of IFNAR2 in immune cells has been shown to be associated with a higher risk of COVID-19, likely due to impaired interferon signaling in the blood (34). *IL10RB* codes for IL-10 receptor beta, and its expression correlates with the severity of COVID-19 disease (35).

Thus, both for rs71327024 and rs12482193, ANANASTRA prioritizes the SNPs as causal and provides information on involved TFs, thus revealing molecular mechanisms behind the association of variants and phenotypes.

## DISCUSSION

Because of linkage disequilibrium, associations detected in genome-wide association studies do not immediately implicate specific causal variants. Instead, for each locus implicated, it is possible to find a variant with the highest posterior probability of being causal and a ‘credible set’ of variants that contains causal one(s) with high (usually 95%) probability. Even for large studies having very high statistical power, posterior probability rarely exceeds 50%, and the number of variants in a credible set is often tens and even hundreds. For smaller studies, the posterior probability is even less degenerate and the credible set size becomes even larger. What are the criteria for the optimal choice of SNPs to serve as an input to ANANASTRA? This depends, at least partly, on the aim of the analysis. Suppose the aim is to characterize the role of ASB mechanism in regulation of population variation of a specific trait through ASB enrichment analysis. In that case, one should perhaps concentrate on SNPs with high enough posterior probability. Practically, one may restrict the input to one SNP per significantly associated locus, selecting the SNP with locally strongest association. When the aim is to hypothesize which of the SNPs in a locus is likely causal and acting through the ASB mechanism, one should consider a set of SNPs including a causal variant with high probability, i.e. a 95% credible set. Note that (strong) enrichment is unlikely to be observed in the latter case. This is explained by the fact that many credible sets are large, but only a few variants in a credible set are expected to be causal.

There is another critical feature of ASBs that should be considered when interpreting ANANASTRA results. The ASBs are generally overrepresented in gene regulatory regions, particularly in promoters in the vicinity of transcription start sites (see Figure 3C in (19)). ANANASTRA does not correct for non-randomness of the genomic localization of ASBs, i.e. if the user-supplied set of SNPs is somehow prefiltered using genomic coordinates and/or functional annotations, this might affect the enrichment estimates. Particularly, enrichment with ASBs will be likely observed for an arbitrary list of SNPs if drawn from gene promoter regions.

Finally, a user should allow for the fact that ANANASTRA is built upon systematically reprocessed but heterogeneous ChIP-Seq data, and for different TFs both the number of source experimental data sets and the total count of significant ASB sites vary tenfolds (see the Data subpage on the ANANASTRA website). The same is true for distribution of experiments across the cell types, with most of the ChIP-Seq data coming from immortalized cell lines rather than from normal tissue samples. Thus, missing a significant known ASB site for a TF or a cell type of interest, or a lack of significant enrichment should not be overinterpreted as it is likely related to an incomplete set of reprocessed experimental data or limitations of the underlying ASB calling procedure. Furthermore, ASBs are often shared between TFs, in particular, due to protein-protein interactions and allele-specific chromatin accessibility. Thus in many cases, it could be informative to follow-up ANANASTRA annotation with additional sequence motif analysis of ASBs to look for potentially causal TFs other than those directly listed by ANANASTRA.

## DATA AVAILABILITY

ANANASTRA is a freely accessible web server available at https://ananastra.autosome.org. Brief documentation is available on the Help page of the web server, a detailed interactive tour is accessible at the bottom right corner of each functional page. ANANASTRA has been running since fall 2020 and is compatible with all commonly-used web browsers (Safari, Chrome, Opera, Firefox, and Microsoft Edge). The website is also fully operational from mobile phones and tablets with smaller screen sizes. The underlying data of ANANASTRA is freely available in the ADASTRA database: https://adastra.autosome.org.

## References

[1] Visscher P.M. , Wray N.R., Zhang Q., Sklar P., McCarthy M.I., Brown M.A., Yang J. 10 Years of GWAS discovery: biology, function, and translation. Am. J. Hum. Genet. 2017; 101:5–22. 28686856 10.1016/j.ajhg.2017.06.005 PMC5501872

[2] Boyle A.P. , Hong E.L., Hariharan M., Cheng Y., Schaub M.A., Kasowski M., Karczewski K.J., Park J., Hitz B.C., Weng S. et al. Annotation of functional variation in personal genomes using RegulomeDB. Genome Res. 2012; 22:1790–1797. 22955989 10.1101/gr.137323.112 PMC3431494

[3] Huang D. , Zhou Y., Yi X., Fan X., Wang J., Yao H., Sham P.C., Hao J., Chen K., Li M.J. VannoPortal: multiscale functional annotation of human genetic variants for interrogating molecular mechanism of traits and diseases. Nucleic. Acids. Res. 2022; 50:D1408–D1416. 34570217 10.1093/nar/gkab853 PMC8728305

[4] Kumar S. , Ambrosini G., Bucher P. SNP2TFBS – a database of regulatory SNPs affecting predicted transcription factor binding site affinity. Nucleic. Acids. Res. 2017; 45:D139–D144. 27899579 10.1093/nar/gkw1064 PMC5210548

[5] Vorontsov E.I. , Kulakovskiy I.V., Khimulya G., Nikolaeva D.D., Makeev V.J. PERFECTOS-APE - Predicting Regulatory functional effect of SNPs by approximate P-value estimation. Proceedings of the International Conference on Bioinformatics Models, Methods and Algorithms. 2015; Lisbon, Portugal SCITEPRESS - Science and and Technology Publications 102–108.

[6] Boytsov A. , Abramov S., Makeev V.J., Kulakovskiy I.V. Positional weight matrices have sufficient prediction power for analysis of noncoding variants. F1000Research. 2022; 11:33. 10.12688/f1000research.75471.1 PMC9237556 35811788

[7] Penzar D.D. , Zinkevich A.O., Vorontsov I.E., Sitnik V.V., Favorov A.V., Makeev V.J., Kulakovskiy I.V. What do neighbors tell about you: the local context of cis-regulatory modules complicates prediction of regulatory variants. Front. Genet. 2019; 10:1078. 31737053 10.3389/fgene.2019.01078 PMC6834773

[8] Zhou J. , Troyanskaya O.G. Predicting effects of noncoding variants with deep learning–based sequence model. Nat. Methods. 2015; 12:931–934. 26301843 10.1038/nmeth.3547 PMC4768299

[9] Melnikov A. , Murugan A., Zhang X., Tesileanu T., Wang L., Rogov P., Feizi S., Gnirke A., Callan C.G., Kinney J.B. et al. Systematic dissection and optimization of inducible enhancers in human cells using a massively parallel reporter assay. Nat. Biotechnol. 2012; 30:271–277. 22371084 10.1038/nbt.2137 PMC3297981

[10] Kwasnieski J.C. , Mogno I., Myers C.A., Corbo J.C., Cohen B.A. Complex effects of nucleotide variants in a mammalian cis-regulatory element. Proc. Natl. Acad. Sci. 2012; 109:19498–19503. 23129659 10.1073/pnas.1210678109 PMC3511131

[11] Kheradpour P. , Ernst J., Melnikov A., Rogov P., Wang L., Zhang X., Alston J., Mikkelsen T.S., Kellis M. Systematic dissection of regulatory motifs in 2000 predicted human enhancers using a massively parallel reporter assay. Genome Res. 2013; 23:800–811. 23512712 10.1101/gr.144899.112 PMC3638136

[12] Yan J. , Qiu Y., Ribeiro dos Santos A.M., Yin Y., Li Y.E., Vinckier N., Nariai N., Benaglio P., Raman A., Li X. et al. Systematic analysis of binding of transcription factors to noncoding variants. Nature. 2021; 591:147–151. 33505025 10.1038/s41586-021-03211-0 PMC9367673

[13] Chen J. , Rozowsky J., Galeev T.R., Harmanci A., Kitchen R., Bedford J., Abyzov A., Kong Y., Regan L., Gerstein M. A uniform survey of allele-specific binding and expression over 1000-Genomes-Project individuals. Nat. Commun. 2016; 7:11101. 27089393 10.1038/ncomms11101 PMC4837449

[14] Shi W. , Fornes O., Mathelier A., Wasserman W.W. Evaluating the impact of single nucleotide variants on transcription factor binding. Nucleic. Acids. Res. 2016; 44:10106–10116. 27492288 10.1093/nar/gkw691 PMC5137422

[15] Cavalli M. , Baltzer N., Umer H.M., Grau J., Lemnian I., Pan G., Wallerman O., Spalinskas R., Sahlén P., Grosse I. et al. Allele specific chromatin signals, 3D interactions, and motif predictions for immune and b cell related diseases. Sci. Rep. 2019; 9:2695. 30804403 10.1038/s41598-019-39633-0 PMC6389883

[16] de Santiago I. , Liu W., Yuan K., O’Reilly M., Chilamakuri C.S.R., Ponder B.A.J., Meyer K.B., Markowetz F. BaalChIP: bayesian analysis of allele-specific transcription factor binding in cancer genomes. Genome Biol. 2017; 18:39. 28235418 10.1186/s13059-017-1165-7 PMC5326502

[17] Chakraborty M. , Das R.K., Samal S., Das S., Alone D.P. Fuchs endothelial corneal dystrophy associated risk variant, rs3768617 in LAMC1 shows allele specific binding of GFI1B. Gene. 2022; 817:146179. 35031421 10.1016/j.gene.2021.146179

[18] Korbolina E.E. , Bryzgalov L.O., Ustrokhanova D.Z., Postovalov S.N., Poverin D.V., Damarov I.S., Merkulova T.I. A panel of rSNPs demonstrating allelic asymmetry in both chip-seq and RNA-seq data and the search for their phenotypic outcomes through analysis of DEGs. Int. J. Mol. Sci. 2021; 22:7240. 34298860 10.3390/ijms22147240 PMC8303726

[19] Abramov S. , Boytsov A., Bykova D., Penzar D.D., Yevshin I., Kolmykov S.K., Fridman M.V., Favorov A.V., Vorontsov I.E., Baulin E. et al. Landscape of allele-specific transcription factor binding in the human genome. Nat. Commun. 2021; 12:2751. 33980847 10.1038/s41467-021-23007-0 PMC8115691

[20] Sherry S.T. dbSNP: the NCBI database of genetic variation. Nucleic. Acids. Res. 2001; 29:308–311. 11125122 10.1093/nar/29.1.308 PMC29783

[21] Schneider V.A. , Graves-Lindsay T., Howe K., Bouk N., Chen H.-C., Kitts P.A., Murphy T.D., Pruitt K.D., Thibaud-Nissen F., Albracht D. et al. Evaluation of GRCh38 and de novo haploid genome assemblies demonstrates the enduring quality of the reference assembly. Genome Res. 2017; 27:849–864. 28396521 10.1101/gr.213611.116 PMC5411779

[22] Berisa T. , Pickrell J.K. Approximately independent linkage disequilibrium blocks in human populations. Bioinformatics. 2015; 32:283–285. 26395773 10.1093/bioinformatics/btv546 PMC4731402

[23] Farh K.K.-H. , Marson A., Zhu J., Kleinewietfeld M., Housley W.J., Beik S., Shoresh N., Whitton H., Ryan R.J.H., Shishkin A.A. et al. Genetic and epigenetic fine mapping of causal autoimmune disease variants. Nature. 2015; 518:337–343. 25363779 10.1038/nature13835 PMC4336207

[24] International Inflammatory Bowel Disease Genetics Consortium Huang H. , Fang M., Jostins L., Umićević Mirkov M., Boucher G., Anderson C.A., Andersen V., Cleynen I., Cortes A. et al. Fine-mapping inflammatory bowel disease loci to single-variant resolution. Nature. 2017; 547:173–178. 28658209 10.1038/nature22969 PMC5511510

[25] Goldberg R. , Clough J.N., Roberts L.B., Sanchez J., Kordasti S., Petrov N., Hertweck A., Lorenc A., Jackson I., Tasker S. et al. A crohn's Disease-associated IL2RA enhancer variant determines the balance of t cell immunity by regulating responsiveness to IL-2 signalling. J. Crohns Colitis. 2021; 15:2054–2065. 34120187 10.1093/ecco-jcc/jjab103 PMC8684452

[26] Schwartz A.M. , Demin D.E., Vorontsov I.E., Kasyanov A.S., Putlyaeva L.V., Tatosyan K.A., Kulakovskiy I.V., Kuprash D.V. Multiple single nucleotide polymorphisms in the first intron of the IL2RA gene affect transcription factor binding and enhancer activity. Gene. 2017; 602:50–56. 27876533 10.1016/j.gene.2016.11.032

[27] Nojima M. , Huang Y., Tyagi M., Kao H.-Y., Fujinaga K. The positive transcription elongation factor b is an essential cofactor for the activation of transcription by myocyte enhancer factor 2. J. Mol. Biol. 2008; 382:275–287. 18662700 10.1016/j.jmb.2008.07.017 PMC4118929

[28] COVID-19 Host Genetics Initiative Mapping the human genetic architecture of COVID-19. Nature. 2021; 600:472–477. 34237774 10.1038/s41586-021-03767-x PMC8674144

[29] Wein A.N. , McMaster S.R., Takamura S., Dunbar P.R., Cartwright E.K., Hayward S.L., McManus D.T., Shimaoka T., Ueha S., Tsukui T. et al. CXCR6 regulates localization of tissue-resident memory CD8 t cells to the airways. J. Exp. Med. 2019; 216:2748–2762. 31558615 10.1084/jem.20181308 PMC6888981

[30] Dai Y. , Wang J., Jeong H.-H., Chen W., Jia P., Zhao Z. Association of CXCR6 with COVID-19 severity: delineating the host genetic factors in transcriptomic regulation. Hum. Genet. 2021; 140:1313–1328. 34155559 10.1007/s00439-021-02305-z PMC8216591

[31] Stikker B. , Stik G., Hendriks R.W., Stadhouders R. Severe COVID-19 associated variants linked to chemokine receptor gene control in monocytes and macrophages immunology. 2021; bioRxiv doi:11 June 2021, preprint: not peer reviewed 10.1101/2021.01.22.427813. PMC9009160 35421995

[32] Patterson B.K. , Guevara-Coto J., Yogendra R., Francisco E.B., Long E., Pise A., Rodrigues H., Parikh P., Mora J., Mora-Rodríguez R.A. Immune-Based prediction of COVID-19 severity and chronicity decoded using machine learning. Front. Immunol. 2021; 12:700782. 34262570 10.3389/fimmu.2021.700782 PMC8273732

[33] Merad M. , Martin J.C. Pathological inflammation in patients with COVID-19: a key role for monocytes and macrophages. Nat. Rev. Immunol. 2020; 20:355–362. 32376901 10.1038/s41577-020-0331-4 PMC7201395

[34] Schmiedel B.J. , Rocha J., Gonzalez-Colin C., Bhattacharyya S., Madrigal A., Ottensmeier C.H., Ay F., Chandra V., Vijayanand P. COVID-19 genetic risk variants are associated with expression of multiple genes in diverse immune cell types. Nat. Commun. 2021; 12:6760. 34799557 10.1038/s41467-021-26888-3 PMC8604964

[35] Voloudakis G. , Hoffman G., Venkatesh S., Lee K.M., Dobrindt K., Vicari J.M., Zhang W., Beckmann N.D., Jiang S., Hoagland D. et al. IL10RB as a key regulator of COVID-19 host susceptibility and severity genetic and genomic medicine. 2021; medRxiv doi:02 June 2021, preprint: not peer reviewed 10.1101/2021.05.31.21254851.

